# The Role of Small Noncoding RNA in DNA Double-Strand Break Repair

**DOI:** 10.3390/ijms21218039

**Published:** 2020-10-28

**Authors:** Iwona Rzeszutek, Gabriela Betlej

**Affiliations:** 1 Institute of Biology and Biotechnology, Department of Biotechnology, University of Rzeszow, Pigonia 1, 35-310 Rzeszow, Poland; 2 Institute of Physical Culture Studies, College of Medical Sciences, University of Rzeszow, 35-310 Rzeszow, Poland; gbetlej@ur.edu.pl

**Keywords:** noncoding RNA, double-strand breaks (DSBs), DNA repair, DNA damage response (DDR)

## Abstract

DNA damage is a common phenomenon promoted through a variety of exogenous and endogenous factors. The DNA damage response (DDR) pathway involves a wide range of proteins, and as was indicated, small noncoding RNAs (sncRNAs). These are double-strand break-induced RNAs (diRNAs) and DNA damage response small RNA (DDRNA). Moreover, RNA binding proteins (RBPs) and RNA modifications have also been identified to modulate diRNA and DDRNA function in the DDR process. Several theories have been formulated regarding the synthesis and function of these sncRNAs during DNA repair; nevertheless, these pathways’ molecular details remain unclear. Here, we review the current knowledge regarding the mechanisms of diRNA and DDRNA biosynthesis and discuss the role of sncRNAs in maintaining genome stability.

## 1. Introduction

During life, all cells in our body are continuously challenged by factors that induce damages in our DNA. Such factors are commonly present in our environment and can be both exogenous and endogenous to the cell [1,2,3]. Exogenous sources of DNA damage include: an ultraviolet light (UV), ionizing radiation (IR) and X-ray [4], while endogenous DNA damage often arises as a product or by-product of cells’ own metabolism [5]. Additionally, DNA damage can also occur during the replication or transcription process. Interestingly, these processes can also occur concomitantly, creating opportunities for either cooperation or conflict [6,7]. The resulting DNA lesions (nucleotide adducts, inter-strand cross-links or single-/double-strand breaks (SSBs/DSBs)) created by these factors are very dangerous and if left unrepaired or repaired incorrectly can lead to: (a) mutations, (b) chromosomal rearrangements, (c) aberrant DNA repair gene expression profiles, all of each contribute to cancer [4].

To counteract these lesions and preserve genome stability, cells evolved a molecular system that detects damaged DNA, signals their presence, promotes their repair as well as initiates signaling pathways that impact a wide range of cellular processes. These actions are collectively known as the DNA-damage response (DDR) [3,4,8]. In fact, cells have developed a number of pathways, each of which recognizes and repairs specific types of damage that occur to DNA. Typically, DSBs are repaired by two main mechanisms: homologous recombination (HR) or an error-prone mechanism known as classical non-homologous end joining (C-NHEJ) [9,10]. In addition, alternative error-prone mechanisms, namely alternative end joining (alt-EJ) and single-strand annealing (SSA) are used [11]. Large nucleotide adducts are repaired by nucleotide excision repair (NER), while individual or small base lesions are repaired by base-excision repair (BER) [10].

Previously it has been thought that DDR involves only enzymatic reactions carried out by proteins facilitating signaling as well as the repair process. Interestingly, a number of reports have implicated the function of noncoding RNA (ncRNA) in DDR [12,13,14], which we would like to discuss here in more detail.

## 2. An Overview of DNA Double-Strand Break Repair Mechanisms

Nuclear DNA, the storage of life information, is undoubtedly the most precious component of a cell, therefore needs to be protected from any lesions, especially from DSBs, which are one of the most harmful forms of DNA damage. Once a DSB is present in the genome, the cell initiates the DDR process of trying to repair the lesion [3,15]. However, if this is inauspicious the cell enters apoptosis [15]. As mentioned previously, these lesions can be repaired either by C-NHEJ, which is a fast, nonspecific pathway, or by HR, a slower, but higher fidelity process [15]. HR uses the sister chromatid as a template during G2 and S-phase for repair (the exception being HR repairing DSB in the rDNA [16]), while the C-NHEJ pathway is predominantly used by the cell during the G1 phase, although it can occur throughout most of the cell cycle [15].

Activation of the cellular response in both pathways starts with the recruitment of proteins that are able to recognize the damaged DNA [17,18,19,20,21,22]. After the recognition of DNA breaks by the sensor proteins, three key phosphatidylinositol 3-kinase like proteins (PIKs): Ataxia Telangiectasia Mutated (ATM), DNA-dependent protein kinase catalytic subunit (DNA-PKcs), and Ataxia Telangiectasia and RAD3 related-protein (ATR) are activated [3]. Once activated, they phosphorylate many downstream targets, including histone variant H2A.X on Ser139 [23]. In case H2A.X gets phosphorylated, it accumulates around DSB sites (up to 1 Mb away from the break site) [24,25]. This modification, known as γH2A.X, provides a scaffold for the amplification of DDR signaling as well as DNA repair [24]. The recruitment of a Mediator of DNA damage checkpoint protein 1 (MDC1) to γH2AX facilitates the activation of the E3-ubiquitin ligases RNF8 and RNF168. This initiates a modification cascade resulting in polyubiquitination of the H1-linker histone and H2A [26,27,28]. Ubiquitination signals the recruitment P53-binding protein 1 (53BP1) and breast cancer type 1 protein (BRCA1), which then, by the opposing activities, govern the repair pathway choice. Binding of 53BP1 to the break stimulates the C-NHEJ, whereas BRCA1 promotes the HR pathway [29].

C-NHEJ occurs via sensing and binding of the Ku heterodimer composed of Ku70-Ku80 subunits to the DSB [30]. Once the Ku heterodimer is bound to the DSB ends, it recruits other downstream factors, such as DNA-PKcs [31,32], X-ray cross-complementing protein 4 (XRCC4) [30,33,34], DNA Ligase IV [31,33], XRCC4-like factor (XLF) [35], Artemis [36] and Aprataxin-and-PNK-like factor (APLF) [37,38,39] that process and ligate the ends to repair the break. If not repaired by C-NHEJ, DSB is fixed by HR which is initiated by the nucleolytic degradation of the 5′ terminated strands in a process termed end resection. End resection occurs via a two-step mechanism involving partially redundant nucleases Sae2/CtIP, Mre11-Rad50-Xrs2/Nbs1 (MRX/N), Exo1, and/or Dna2. Generated in this process, the 3′ single-strand overhangs get coated in a RAD51 protein filament to catalyze the homologous pairing and exchange of DNA strands [9].

Interestingly, recent studies indicated a new component in the DDR cascade: the noncoding RNA as well as RNA binding proteins which will be discussed in the next sections.

## 3. Noncoding RNA in Double-Strand Break Repair

The term noncoding RNA is commonly used in the case of RNA that does not encode a protein, however, it does not mean that such RNA does not contain any information or possess any function. They are mainly processed from double-stranded RNA (dsRNA) precursors by Dicer or Dicer-like proteins. Next, associated with Argonaute (AGO) or Piwi proteins (a central factor in the RNA-induced silencing complex (RISC)), they regulate the gene expression at the post-transcriptional level or target DNA for excision [40,41]. It turns out that many species evolved processes in which noncoding RNA is involved. For instance, these small, 20–30 nucleotides (nt) ncRNAs are able to silence gene expression through the interaction with mRNA: microRNA (miRNA) and small interfering RNA (siRNA). Similarly, Piwi-interacting RNA (piRNA) and siRNA are involved in epigenetic regulation [42,43]. Moreover, it has been shown that ciliates, a class of unicellular eukaryotes, possess small ncRNAs that are able to target DNA for elimination/excision [41,44,45,46,47]. Recent data also indicated the involvement of small noncoding RNAs in the DDR process [12,13], which we would like to describe in more detail in the following sections.

### 3.1. De Novo RNA Synthesis at DSBs

**Figure 1:**
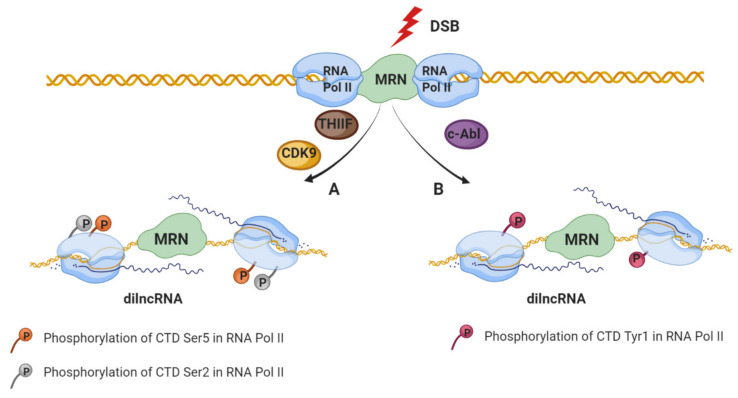
Two models of dilncRNAs production. In response to double-strand breaks (DSB), MRN complex recognizes the DNA damage and recruits RNA Pol II through binding with its RPB1 subunit. (**A**) Damage-induced RNA (dilncRNA) are transcribed by RNA Pol II phosphorylated on the carboxy-terminal domain (CTD) at Serine5 or Serine2 residue that regulates elongation and RNA processing. (**B**) Transcription of dilncRNA is stimulated by c-Abl which is capable of inducing phospho-marks of RNA Pol II predominantly at CTD Tyrosine1.

It is widely known that DDR induces signaling pathways that remodel chromatin in the proximity of DSBs [48,49] causing an inhibitory effect on transcription at nearby promoters in order to avoid collisions or interference of the transcriptional and repair machinery [50,51,52,53,54]. The DSBs’ inhibitory effect of transcription by RNA polymerase II (RNA Pol II) depends on the ATM and/or DNA-PKcs, the chromatin remodeler BRG1, Poly (ADP-ribosyl) polymerase 1 (PARP1), E subunit of the negative elongation factor (NELF-E) as well as on the distance to the DSBs (as far as 1Mb away from the break) [50,51,55,56,57,58]. Despite the well-established transcriptional inhibition described above, the first insights regarding the production of small RNAs in the vicinity of DSBs come from the studies performed on filamentous fungus *Neurospora crassa* [59]. A growing body of evidence shows that transcription occurs at the DSBs (in the absence of promoters) and that the DSBs repair process involves not only DDR proteins, but also RNA that are produced in the proximity of DSBs. Interestingly, studies have shown that de novo transcription occurs at the open DNA ends in vitro and in vivo in yeast and mammalian cancer cells [12,13,60,61,62,63,64]. Specifically, RNA Pol II accumulates at DSB and initiates transcription from the broken ends to produce non-polyadenylated damage-induced long noncoding RNAs (dilncRNAs) (Figure 1) [63]. Importantly, the dependency of dilncRNA synthesis on RNA Pol II was supported by the following observations: increased occupancy of RNAPII around the break sites, mapping analyses of increased transcripts to sequences around the breaks, as well as detection of dilncRNA in native RNA pull-down after the induction of sequence-specific DSB [56,65]. Additionally, DSB-dependent transcription, like promoter associated-transcription, is sensitive to the inhibition of transcription factor IIH (TFIIH) as well as cyclin-dependent kinase 9 (CDK9) by Triptolide and 5,6-Dichloro-1-beta-Ribo-furanosyl Benzimidazole (DRB), respectively, leading to the phosphorylation of the RNA Pol II carboxy-terminal domain (CTD) residue Ser2/5 (Figure 1A) [63,65,66]. Despite the well-established affinity of RNA Pol II to the free DNA [56,67], regulated recruitment of RNA Pol II to the DSB has been recently described [65]. Specifically, c-Abl (nuclear tyrosine kinase with multiple functions in the DDR [68]) stimulate the phospho-marks of RNA Pol II predominantly at CTD Tyr1, at DSBs, which in turn is necessary for dilncRNA transcription (Figure 1B) [65]. Furthermore, the transcription initiation of dilncRNA at the broken DNA is regulated by the MRE11-RAD50-NBS1 (MRN) complex which directly binds to the RNA Pol II (RPB1 subunit) [63]. However, the details regarding the mechanism of transcription initiation at the broken end remain fully uncovered.

Additionally, this lncRNA produced by the transcription from broken ends has also been shown to stimulate the recruitment of factors involved in DSB repair through HR [61]. In the S/G2 cell-cycle phase, when CtIP is phosphorylated, dilncRNA binds to the resected DNA ends forming a DNA:RNA hybrid recognized by BRCA1. This event subsequently stimulates the recruitment of other HR factors, namely breast cancer type 2 (BRCA2), RAD51 [69] and RAD52 [70]. Moreover, it has been shown that BRCA2 controls DNA:RNA hybrid levels at DSBs by interacting with and mediating RNase H2 recruitment to DSBs [69]. Together, these data show that dilncRNAs play a role in the signalling and DSBs repair.

### 3.2. Biogenesis of Small Noncoding RNA in Response to DSBs

**Figure 2:**
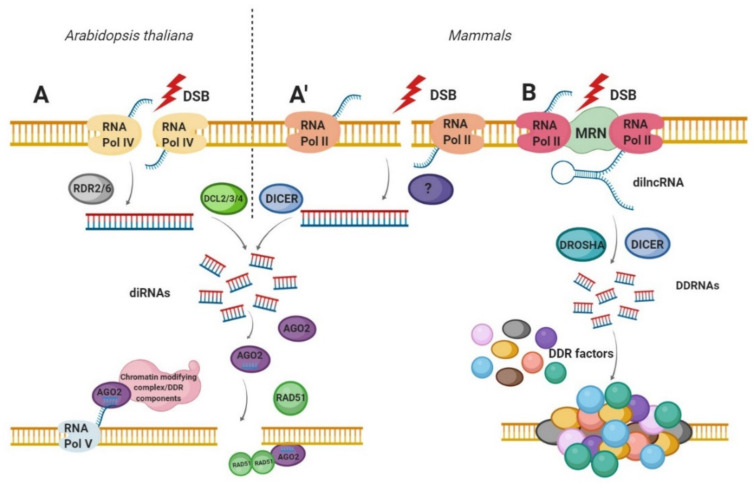
Biogenesis of double-strand break-induced RNA (diRNA) and DNA damage response small RNA (DDRNA). (**A**) diRNAs biogenesis in *Arabidopsis thaliana* and (**A’**) in human cells. After DSB induction, RNA Pol IV (in plants) and RNA Pol II (in humans) transcribe the DNA in the vicinity of DSB to produce the primary transcripts. RDR2 or RDR6 in plants then uses these ssRNA as a template for dsRNA synthesis. In humans, the molecular factors involved in this step have not yet been characterized. Subsequently, dsRNAs are cleaved by DCL2, DCL3 or DCL4 (in plants) or by DICER (in mammals), to produce a pool of 21–24 nt long small RNAs (diRNAs). Next, diRNAs are incorporated into AGO2 proteins leading to the removal of the passenger strand. The AGO2—diRNA complexes act as a guide for DNA damage response (DDR) factors such as RAD51 or chromatin remodelers to the DSB. (**B**) The DDRNAs biogenesis in mammals. After DSB induction, primary sensor of DSBs—MRN complex is bound to the site of damaged DNA. Next, the RNA Pol II synthesizes damage-induced long noncoding RNAs (dilncRNAs) from and towards DNA ends. dilncRNAs are then cleaved by DICER and DOSHA, generating DDRNA that participates in DDR signal amplification.

Excitingly, different groups of scientists have reported a direct link between DNA damage and small noncoding RNA (sncRNA) production. These sncRNAs produced in response to DSBs are termed: double-strand break-induced RNAs (diRNAs) and DNA Damage Response Small RNA (DDRNA) [12,13]. The biogenesis and the function of these sncRNAs rely on specific components of the RNA interference (RNAi) machinery connecting their canonical roles in gene silencing with DDR and genome stability [71]. Specifically, the phosphorylated form of Dicer (p-Dicer) accumulates in the nucleus and is recruited to DSBs where it catalyzes damage-induced RNA processing [72]. Therefore, the loss of RNAi components leads to DNA damage accumulation and increased genome instability in mammals and plants [12,72,73]. The diRNAs and DDRNA biogenesis is discussed in more detail in the next subsections and is illustrated in Figure 2.

#### 3.2.1. Double-Strand Break-Induced RNAs (diRNA)

Double-strand break-induced RNAs (diRNA) are a class of 21–24 nt long small RNAs produced in the close vicinity of DSB in *Arabidopsis thaliana* as well as human cells [12]. Production of those RNAs occurs in an ATR kinase-dependent manner by plant RNA polymerase IV [12,74]. However, DSBs-induced γH2AX or DDR foci formation was not affected in diRNAs depleted cells [12,75], suggesting that those molecules respond in a process independent or downstream of DNA repair factors [75]. diRNAs are generated as a product of Dicer (in human) or Dicer-like (DCL) (in plants) protein activity [12,66,74]. Subsequently, Dicer-processed products are incorporated into AGO2 proteins and act as a guide, based on homology, recruiting DDR factors to DSB [12,14,66]. In mammalian cells, the depletion of Dicer or AGO2 affects the DNA repair process [12]. These data were confirmed by Wang and Goldstein, who have shown that the transfection of oligo-RNAs rescued HR mechanism (RAD51 and BRCA1 foci formation) after Dicer or Drosha downregulation [76]. Moreover, the interaction between RAD51 and AGO2 facilitates the recruitment of RAD51 to single-stranded DNA filaments at the DSBs. Interestingly, diRNA is redundant for RAD51 and AGO2 interaction, however, they are required for RAD51 foci formation at DNA damaged sites in a homology-dependent manner [14]. Additionally, recent data suggest that RAD51 recruitment might need two levels of regulation; besides AGO–RAD51 interaction, the chromatin remodeling level seems to be more crucial [76]. Indeed, Wang and Goldstein [76] reported about diRNA’s ability to increase the recruitment of chromatin remodelers, such as the methyltransferase MMSET and the acetyltransferase Tip60 to the DSB. Surprisingly, it turns out that AGO2 plays a key role in this mechanism, as it is able to bind to the break and cooperate with MMSET and Tip60. Therefore, diRNAs might act as a guide molecule for AGO2 and regulate chromatin remodeling at the DSB site [76]. To further characterize this class of sRNAs in *Arabidopsis* and rice, scientists used clustered regularly interspaced short palindromic repeats (CRISPR)/CRISPR-associated protein 9 (Cas9) system and transcription activator-like effector nucleases (TALEN) technology to trigger site-specific DSBs [74]. These data confirmed previous studies showing that diRNAs strongly accumulate at transcribed transgenes [12,74]. Furthermore, they found that diRNAs are also generated at transgenes that lack a direct repeat, however, diRNAs were not detected from endogenous regions or repetitive sequences [74]. Interestingly, the biogenesis of diRNAs also involves RNA polymerases V as their inactivation increased the diRNAs level, and reduced DNA repair [12,74]. Moreover, RNA dependent RNA polymerase (RDR) 6 and DCL4 are also required for diRNAs synthesis as their mutation impair diRNAs accumulation, however, without affecting DNA repair rate [74]. All this suggests that diRNAs modulate DDR process, however the exact mechanism remains only partially understood.

#### 3.2.2. DNA Damage Response Small RNA (DDRNA)

DNA Damage Response Small RNA (DDRNA) present in mammalian cells, like diRNA are generated in response to DSB, however, the mechanism of their biogenesis is different. The main differences between these two classes of ncRNAs are listed in Table 1. Briefly, RNA Pol II is first recruited to DSB in a MRN-dependent manner [13] to produce previously described dilncRNAs [63]. Synthesis of dilncRNAs occurring from DSB ends in a bidirectional manner [63]. Next, dilncRNAs are cleaved into shorter, 21-nt long DDRNAs by the Dicer and Drosha endoribonucleases [13]. Moreover, dilncRNAs interact with DDRNAs and guide them (through base-pairing) to the damaged DNA, resulting in the DDR foci formation [63].

The first evidence suggesting the potential role of DDRNA in DDR response have been provided by Francia et al. [13]. They indicated that cells treated with RNaseA completely abolished DDR formation and this effect may be rescued by adding chemically− or in vitro−synthesized site-specific DDRNAs or by RNA purified from cells with induced DSBs [13,77]. Moreover, after laser-induced DSBs in Dicer and Drosha-knockdown cells, NBS1 (a part of MRN complex) and γH2AX recruitment were unaffected, suggesting that DNA damage recognition is independent from DDRNAs and the molecules promoting DNA damage signal amplification [13,77].

Recent reports described the presence of DNA:RNA hybrids around the DSBs which are formed in a Drosha-dependent manner [61]. Those hybrids were crucial for proper DNA repair and their degradation through RNaseH1 predisposed cells for DNA damage. Here, the researchers also observed that Drosha was not involved in the early stage of DDR signaling, and the depletion of Drosha did not affect H2AX phosphorylation or ATM recruitment. However, Drosha was bound to DNA damage and required for the redistribution of 53BP1, one of the well-known DDR factors [61]. Furthermore, researchers tried to investigate the production of DDRNA from the vicinity of endogenous genomic DSB regions using next-generation sequencing approach [61]. However, these studies failed to investigate the presence of this class of sncRNAs at any endogenous cut sites [61], which is in conflict with previous reports [13,60,78].

Interestingly, there are more inconsistencies regarding the studies of sncRNAs in the DDR pathway, which we would like to briefly mention. Previously discussed reports suggested the inhibition of HR/NHEJ and DDR foci formation after Drosha or Dicer knockdown [13,61,77,78], as well as in response to antisense oligonucleotides (ASOs)-mediated dilncRNA inactivation [69]. However, the promotion of NHEJ mediated DNA repair and the reduction in HR efficiency was recently observed after stimulating Dicer activity by enoxacin, suggesting that increasing Dicer activity, and in response to this, DDRNAs production, in some cases modulates the accuracy of DSB repair [79]. Additionally, researchers also elucidated the role of DDRNAs in the stimulation of 53BP1 foci formation [63,78,79], one of the key factors involved in choosing the DBS repair pathway by promoting NHEJ and inhibiting HR [80]. The role of 53BP1 in the regulation of DNA repair and the generally prevailing view of competition between NHEJ and HR support the results presented by Gioia et al. [79]. Several research groups abreast about the competition of those mechanisms [81,82], however, recent data supports entwined relationship between HR and NHEJ in repairing DSBs rather than exclusive competition [83]. Taken together, the discussed results demonstrate that DDRNA promotes DSB repair and DDR foci formation, however, it seems circumspect to investigate not only the molecular details of this regulation, but also whether DDRNA, in some part, can participate in the selection of the DNA repair pathway.

#### 3.2.3. Telomeric DNA Damage Response Small RNAs (tDDRNAs)

Interestingly, the production of DDRNs and their longer precursors have also been shown at telomeres. These sncRNAs are transcribed from both strands of deprotected telomeres, hence are called telomeric DDRNAs (tDDRNAs) and telomeric dilncRNAs (tdilncRNAs) [78,84,85]. Similarly, as in DDRNAs biogenesis, silencing of Dicer and Drosha fully abolished tDDRNA production while Drosha-knockdown lead to the accumulation of tdilncRNA [78], suggesting that both the proteins are crucial for tDDRNAs biosynthesis.

Moreover, 53BP1 foci formation in response to DDRNA induction was described in telomeres as well. Despite unaltered total 53BP1 protein concentration, cells treated with IR were sensitive to RNaseA and displayed decreased DDR foci formation. Interestingly, the rescue of foci was possible only by DDRNAs with telomeric sequences. Moreover, cells depleted for Dicer or Drosha were ineffective in 53BP1 foci formation, confirming the role of these two proteins in tDDRNAs generation and DDR response [78]. Additionally, in fibroblasts isolated from patients with Hutchinson–Gilford progeria syndrome (HGPS), characterized by telomeres dysfunction and premature aging, levels of tdilncRNAs and tDDRNAs increased. Further study on a mouse model of HGPS indicated that the concentration of markers of DDR activation such as 53BP1 and ATM were higher compared to wild-type cells. Additionally, using telomeric sequence-specific ASOs, which block both—tDDRNAs and tdilncRNAs, inhibited DDR in telomeres providing further evidence for the regulation of 53BP1 foci formation via DDRNAs [84].

### 3.3. RNA Modification Involved in DNA Damage Response

Another piece of evidence suggesting the involvement of RNA in the DNA repair process has been shown by Xiang et al. [86]. The authors detected the presence of N6-methyladenosine (m6A) at DNA damage sites in response to ultraviolet (UV) laser irradiation within a short period (2 min). RNA m6A is known to be regulated by the methyltransferase METTL3 (methyltransferase-like 3) [87] and the demethylase FTO (fat mass and obesity-associated protein) [88]. The absence of METTL3 catalytic activity leads to delays in the repair of UV-induced cyclobutane pyrimidine dimers (CPD). METTL3 absence also affects the localization of DNA polymerase κ (Pol κ) at DNA damaged sites. Taken together, authors demonstrated the importance of m6A modification of RNA in the UV-responsive DNA damage response [86].

A few years later, Svobodova Kovarikova et al. [89] confirmed the observations made by Xiang et al., showing the accumulation of 6mA RNA modification at the DNA lesions explaining that this effect could be a consequence of the coregulatory function of METTL-like enzymes or diffusion of m6A RNA to UVA-damaged chromatin [86]. They further observed that m6A RNAs being present in the vicinity of DSBs likely concerns noncoding RNAs, rather than mRNA or rRNA [89]. Additionally, they indicated that inhibition of Suv4-20h1/h2 methyltransferases, responsible for H4K20me2/me3 which is recognized by the 53BP1 protein (NHEJ component) did not affect m6A RNAs at the DNA lesions. These data support the results obtained by Xiang et al. showing that m6A RNA is likely playing a role in the NER mechanism [86,89].

Moreover, recent studies performed by Zhang et al. [90] have shown that METTL3 is phosphorylated by ATM in response to DSBs and subsequently recruited to the DNA lesions [90]. This led to the m6A modification on the DNA damage-associated RNA, recognized and protected by the RNA m6A reader protein: YTH domain-containing protein 1 (YTHDC1). Consequently, the modified RNA forms DNA:RNA hybrids at DSBs which stimulate proteins involved in DDR, such as BRCA1 and RAD51.

## 4. Conclusions and Outlook

For a long time, RNA was considered only as a DNA working photocopy, produced in the process of transcription to generate functional proteins. However, the discovery of noncoding RNAs initiated a series of studies to explore their potential role in various biological processes. In this review, we have discussed the potential function of small noncoding RNAs associated with DSBs in the maintenance of genome stability. We have mainly focused on the mechanisms of diRNAs’ and DDRNAs’ biosynthesis and their role in the DDR pathway. We have also mentioned the process of de novo RNA synthesis at the DSBs. Based on these studies, it is becoming clear that RNA may be employed by the DDR process, as many of DDR factors are required to process RNA in response to DSBs. For instance, DNA-PKcs, RAD52, BRCA1, 53BP1 interact with RNA as well as DNA:RNA hybrids, which are key structures required for DDR progression [70,91,92]. An attractive hypothesis that supports the function of RNA in this phenomenon is that the dilncRNA forms DNA:RNA hybrids that serve a platform to stimulate repair agents [69,70]. Besides, many RNAi components are ostensibly involved in DDR. Although it is agreed that Dicer, Drosha and AGO2 have a role in the DNA repair process, their mechanism remains intangible [12,13,14,61,76,77]. Moreover, RNA modification has been identified as an important biological process involved in DNA damage response [86,89,90].

While we have a basic understanding of how sncRNAs participate in the maintenance of genome stability, numerous outstanding questions remain open. For instance, dilncRNAs’ association to the transcriptionally active chromatin, the timing of dilncRNAs’ biogenesis, or dilncRNAs’ regulation of DDR response remains elusive. It would also be interesting to investigate the molecular details of the regulation of the DDR process by sncRNAs as well as to discern whether DDRNA, in some part, can participate in the selection of the DNA repair pathway. Additionally, 6mA RNA modification’s involvement in the DDR process that has been recently identified require further studies. For instance, it would be interesting to investigate the possible role of other modifications, such as N1-methyladenosine (m1A) and N5-methylcytosine (m5C), in the DNA repair process. Further investigations of sncRNA, RNA binding proteins (RBPs) as well as RNA modifications after DSBs will provide a deeper understanding of mechanisms that govern the maintenance of genome stability. This, in addition, can further help the research community to develop novel RNA therapeutic strategies in the treatment of diseases linked to DNA damage or genetic disorders.

## Figures and Tables

**Table 1:** Differences between diRNA and DDRNA.

	diRNA	DDRNA
Synthesis distance from DSB	Synthesized even from hundred bases away from DNA ends	Synthesized from the sequence that flank DSB
Have the sequence of the damaged locus	No	Yes
Lengths	21–24 nt	20–35 nt
Precursors	Not found	dilncRNAs
Necessary for accumulation	RNA Pol IV, RDR6, AGO2, ATR, Dicer or DCL	Dicer, Drosha, RNA Pol II
Dicer and Drosha dependent	Dicer- or DCL-dependent	Both
Role in DDR	Together with AGO2 promote HR, NHEJ and chromatin structures rearrangements	Induce DDR foci formation and modulate HR repair
